# The early life gut microbiota and atopic disease

**DOI:** 10.1186/1710-1492-10-S2-A63

**Published:** 2014-12-18

**Authors:** Leah T Stiemsma, Marie-Claire Arrieta, Pedro A Dimitriu, Lisa Thorson, Sophie Yurist, Rollin Brandt, Diana L Lefebvre, Padmaja Subbarao, Piush Mandhane, Allan Becker, Malcolm Sears, Tobias Kollmann, William W Mohn, B Brett Finlay, Stuart E Turvey

**Affiliations:** 1 Department of Microbiology & Immunology, University of British Columbia, Vancouver, British Columbia, V6T 1Z4, Canada; 2 The Child and Family Research Institute, Vancouver, British Columbia, V4Z 4H4, Canada; 3 Michael Smith Laboratories, University of British Columbia, Vancouver, British Columbia, V6T 1Z4, Canada; 4 Department of Statistics, University of British Columbia, Vancouver, British Columbia, V6T 1Z4, Canada; 5 St. Joseph’s Healthcare, Hamilton, Ontario, L8N 4A6, Canada; 6 Department of Medicine, McMaster University, Hamilton, Ontario, L8S 4L8, Canada; 7 Department of Pediatrics, University of Toronto, Toronto, Ontario, M5S 2J7, Canada; 8 Hospital for Sick Children, Toronto, Ontario, M5G 1X8, Canada; 9 Department of Pediatrics, University of Alberta, Edmonton, Alberta, T6G 2R3, Canada; 10 School of Public Health, University of Alberta, Edmonton, Alberta, T6G 2R3, Canada; 11 Department of Pediatrics and Child Health, University of Manitoba, Winnipeg, Manitoba, R3T 2N2, Canada; 12 Department of Pediatrics, University of British Columbia, Vancouver, British Columbia, V6T 1Z4, Canada; 13 Department of Biochemistry and Molecular Biology, University of British Columbia, Vancouver, British Columbia, V6T 1Z4, Canada

## Background

Asthma is the most prevalent of all childhood diseases and accounts for the majority of hospitalizations and school absences in children [1]. Current mouse model research has identified the early life gut microbiota as a potential therapeutic target for the prevention of asthma and atopic diseases [2-4]. We hypothesize that the early life gut microbiota could play a similar preventative role against atopic disease development in humans.

## Methods

1262 children enrolled in the Canadian Healthy Infant Longitudinal Development (CHILD) Study with complete skin prick test and wheeze data at one year were grouped into four clinically relevant phenotypes: atopy + wheeze, atopy only, wheeze only, and control. Bacterial 16S rDNA from 3-month and 1-year stool samples of 319 children in these four phenotypes was extracted, amplified, and subjected to high throughput Illumina sequencing. Quantitative polymerase chain reaction (qPCR) and short chain fatty acid (SCFA) analysis were also conducted on 44 children in the two extreme phenotypes (atopy + wheeze vs. control).

## Results

16S sequence analysis of our sample cohort (319 subjects) identified bacterial populations that differed in abundance in the atopy + wheeze group at 3-months of age but not at 1-year of age. Additionally, significant changes in the abundance of certain bacterial genera were found in the atopy + wheeze group when compared to controls by qPCR at 3-months of age only. Changes in stool short chain fatty acid production between the atopy + wheeze group and the control group were also observed at 3-months of age only.

## Conclusions

Shifts in the relative abundance of certain gut bacterial populations and differences in the levels of stool SCFAs before 3-months of age are associated with atopy and wheeze at one year of age.
